# DEMAND-ABILITIES FIT AND PRESTIGE INFLUENCE OLDER WORKERS’ INTEREST IN ALTERNATIVE JOBS

**DOI:** 10.1093/geroni/igad104.3260

**Published:** 2023-12-21

**Authors:** Nathan Baker, Jenna Van Fossen, Amy Schuster, Chu-Hsiang Chang, J Kevin Ford

**Affiliations:** Michigan State University, East Lansing, Michigan, United States; Clemson University, Clemson, South Carolina, United States; Clemson University, Clemson, South Carolina, United States; Michigan State University, East Lansing, Michigan, United States; Michigan State University, East Lansing, Michigan, United States

## Abstract

With the increasing displacement of workers due to technological innovations, many older workers may need or want to make career transitions. Therefore, it is critical to understand the factors that influence older workers acceptance of potential alternative careers. In this study, long-distance truck drivers (n=202), a profession at-risk of technological disruption, completed an online survey where they rated six alternative transition occupations (e.g., machine setting, operator and tender, cargo and freight agent) to understand what makes them interested in alternate jobs. Using multilevel modeling (with occupations nested in participants), we evaluated the role of perceptions of demand-abilities fit and prestige, as moderated by age, on interest in alternative careers. Demand-abilities fit and job prestige were positively associated with rated job appeal, which was positively associated with interest in the job as a transition occupation. Older drivers, relative to younger truck drivers, found job prestige to be more appealing, which appeared to make them more interested in transitioning into these jobs. Our results extend person-environment fit and socio-emotional selectivity theory by showcasing how age relates to evaluations of occupations for career transitions, an area that is rising in importance given the increases in an aging workforce and potential disruptions from automation. Results carry practical importance for preparing the workforce for career disruptions given automation by identifying qualities that may shape workers’ willingness to transition, and identifying how these qualities differ based on age.

